# Accuracy of compact-stencil interpolation algorithms for unstructured mesh finite volume solver

**DOI:** 10.1016/j.heliyon.2021.e06875

**Published:** 2021-04-23

**Authors:** Adek Tasri, Anita Susilawati

**Affiliations:** a Mechanical Engineering Department, Universitas Andalas, Padang 2135, Indonesia; b Mechanical Engineering Department, Universitas Riau, Pekanbaru 28293, Indonesia

**Keywords:** Finite volume, Interpolation algorithm, Unstructured mesh, Accuracy comparison

## Abstract

This study considers the accuracy of cell-to-face centre interpolation of convected quantities in unstructured finite volume meshes with cell-centred storage of variables. The accuracy of the interpolation algorithms were tested in isolation using ideal data to determine their numerical accuracy on both standard and artificially distorted meshes. It was found that the formally second- and third-order accurate interpolations based on one-dimensional interpolation along the line connecting the cells to the right and left of the face under consideration only have first-order accuracy in standard unstructured mesh, and less than first-order accuracy in distorted unstructured mesh. *L1* interpolation errors in the distorted unstructured mesh are greater than in standard unstructured mesh. The order of accuracy and *L1* errors can be improved by applying spatial corrections. The formally second-order accurate multi-dimensional interpolations tested in this study that are not based on one-dimensional interpolation along lines connecting the neighbour cells have first-order accuracy in both standard and distorted unstructured mesh. Linear interpolation between end vertices produces greatest *L1* error in standard mesh; polynomial interpolation, linear interpolation between cell centres and standard QUICK produce the greatest *L1* error in distorted mesh. Spatially correct QUICK, spatially correct linear interpolation between cell centres, Laplacian interpolation to face centres, and Taylor series expansion about an upstream cell produce the smallest *L1* error in both standard and distorted mesh. Based on accuracy and the simplicity of implementation, Taylor series expansion about an upstream cell is the best choice for use in unstructured mesh.

## 1 Introduction

The finite volume solvers that used unstructured and cell centre storage mesh have gained high popularity in the solution of heat and fluids flow problems because the methods can efficiently model real engineering fluids flow problems that have complex geometric boundaries. The approach have been used in some commercial CFD codes, such as Ansys FLUENT and Ansys CFX.

Unstructured meshes typically use triangular cells for two-dimensional problems, allowing great flexibility in efficiently modelling complex boundaries, and in enabling localized grid refinement. The cost of this flexibility is that the familiar locally one-dimensional operators for differentiation and interpolation are no longer applicable and must be replaced by new multi-dimensional algorithms, the properties of which are less well-established, although the algorithms have been widely reported in the literature. A number of researchers have attempted to use one-dimensional interpolation on multi-dimensional meshes, including Croft [1]. Weiss *et al.* [2] and Tasri [3] developed interpolation schemes to reduce the effect of non-orthogonality for momentum interpolation algorithms used in unstructured mesh. Barth and Jespersen [4] introduced an upwind biased scheme with basic cell face properties calculated using cell properties at an upstream cell centre. The scheme was derived using Taylor series expansion of flow variables around the cell centre. Leonard [5] introduced the Quadratic Upstream Interpolation Convective Kinetics (QUICK) scheme for interpolation of properties from cell centre to cell face on structured mesh. The upwind bias and QUICK scheme were later corrected by other authors to improve the accuracy and stability in unstructured grid applications. Darwish and Moukalled [6] and Tasri [7] extended the QUICK scheme for unstructured mesh application. Kim and Choi [8] improved the central differencing scheme by adding a correction to the central differencing equation. Frink [9] calculated the face value of flow variables by averaging face vertices values; the vertices values were reconstructed from cell values around each vertex. Wang and Ren [10] used spline interpolation to solve Euler and Navier-Stokes equations. Using high-speed flow test cases, they found that the hybrid WENO and spline reconstructions were more accurate than the MUSCL scheme. Katta et al. [11] developed a high-order 1-D interpolation procedure that combines a cubic and a quadratic interpolations to address the discontinuous edges of the cubed-sphere grid. Most recently, Vakilipour et al. [12] developed physical influence upwind interpolation schemes for linking pressure and velocity fields in incompressible flow solutions.

These interpolation algorithms have advantages and disadvantages; a comparison is necessary to determine the best algorithm. Lehnhauser and Schafer [13] compared the accuracy of a central differencing scheme and a Taylor series expansion-based scheme; they found that the Taylor series outperformed the central differencing scheme. Fakuchi [14] also compared the performance of interpolation schemes in solving Poisson equations. McBride *et al.* [15] compared vertex-based discretisation using the shape function for the distribution of variables in volume control with cell centre discretisation.

Some researchers have compared interpolation methods using certain case studies; other researchers have also compared interpolation methods with different case studies. The best interpolation method among popular existing methods is still uncertain because the techniques are not compared using the same test case.

In this study, several popular interpolation methods used in commercial numerical software and a newly developed method were compared to determine the interpolation method that produces the smallest error for an unstructured mesh finite volume solver. The Kovasznay's model of viscouse flow [16] and potential flow past a circular cylinder [17] were used as analytical solution benchmarks.

## 2 Finite volume discretisation of governing equation

**Figure 1:**
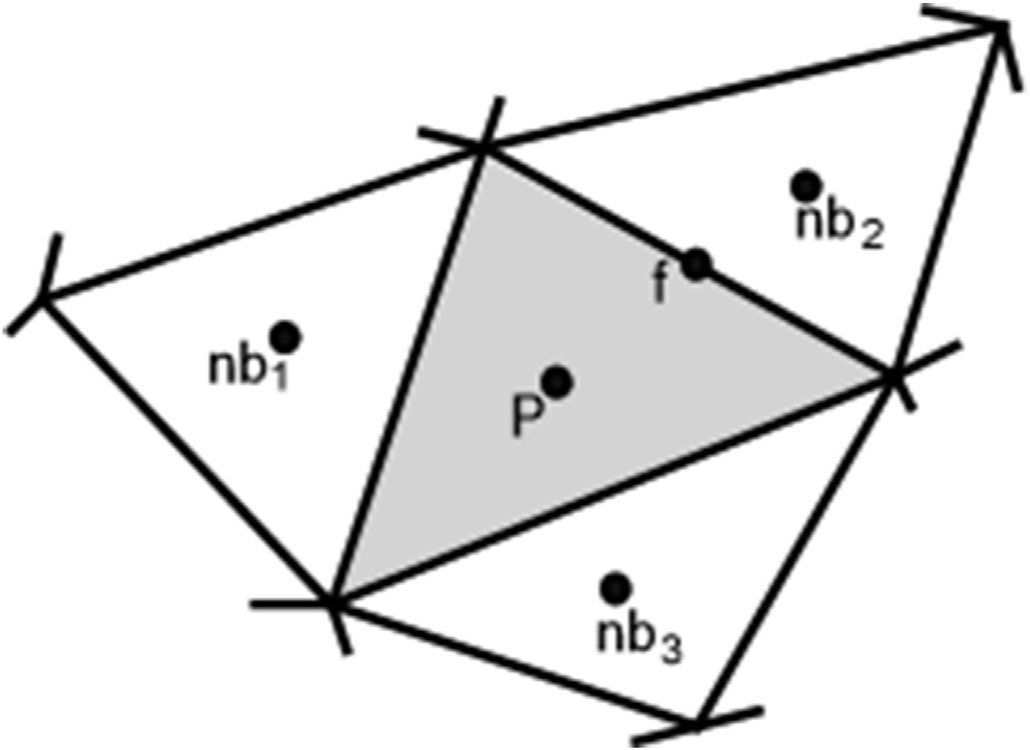
2-D cell and its immediate neighbours.

The governing equations of 2-D incompressible laminar steady flow are incompressible continuity and the Navier-Stokes equation:

(1) $$\frac{\partial u}{\partial x}+\frac{\partial v}{\partial y}=0$$

(2) $$\begin{array}{l} \frac{\partial \rho uu}{\partial x}+\frac{\partial \rho vu}{\partial y}=\frac{\partial }{\partial x}\left(\mu \frac{\partial u}{\partial x}\right)+\frac{\partial }{\partial y}\left(\mu \frac{\partial u}{\partial y}\right)-\frac{\partial p}{\partial x}\\ \frac{\partial \rho uv}{\partial x}+\frac{\partial \rho vv}{\partial y}=\frac{\partial }{\partial x}\left(\mu \frac{\partial v}{\partial x}\right)+\frac{\partial }{\partial y}\left(\mu \frac{\partial v}{\partial y}\right)-\frac{\partial p}{\partial y} \end{array}$$

u and v, represent the xcomponent of velocity and the ycomponent of velocity. μ and p represent the viscosity coefficient and the pressure, respectively. Finite volume discretisation of the Navier-Stokes equation is accomplished by integrating the equation using the Gauss divergence theorem, yielding:

(3) $$\int_{A}\left(\rho \varphi \vec{V}\right)\cdot \vec{n}dA=\int_{A}\left(\mu \nabla \varphi \right)\cdot \vec{n}dA+\int_{\Omega }qd\Omega$$

where φ represents the x and ycomponents of velocity; $\vec{V}$is a velocity vector; q, A, and Ω are the source term, the surface, and the volume of finite volume grid cells, respectively; $\vec{n}$ is a unit vector normal to the surface grid cell. For numerical solution, these equations need to be discretised so that the equations may be applied to the finite volume cells in the solution domain. The equation, for a finite volume cell that has a finite number *f* of identifiable plane faces, may be discretised using second order midpoint rule

(4) $$\sum_{m}{\left(\rho_{f}\varphi_{f}{\vec{V}}_{f}\right)}_{m}\cdot \vec{n}A_{m}=\sum_{m}{\left(\mu_{f}\nabla \varphi_{f}\right)}_{m}\cdot \vec{n}A_{m}+q\Omega$$

where the subscript *f* represents the value of the variables at the face centres and m is index for the faces of the control volume.$A_{m}$ represents the area of face *m*^*th*^ of the control volume. The methods for obtaining the face value of φ are explained in the next section; the face values of φ may be written in terms of cell centroid value for the current cell P and the values for neighbour cells nb immediately adjacent. The cell *P* and neighbour cells *nb* are defined in Figure 1. Eq. (4) can be simplified into the following form:

(5) $$a_{P}\varphi_{P}=\sum_{nb}a_{nb}\varphi_{nb}+S_{\varphi_{P}}$$

$\varphi_{P}$ and $\varphi_{nb}$ are cell centroid value of φ for current cell P and neighbour cell *nb*;$S_{\varphi_{P}}$is a source term that lump all terms not included in the first and second terms of Eq. (5); $a_{P}$ and $a_{nb}$ are determined by the equation for the face values of φ and ∇φ.

Follow the SIMPLE algorithm, the continuity equation is discretised in a finite volume cell *P* to form the pressure correction equation [3].

(6) $${\bar{a}}_{p}p^{\prime }=\sum_{nb}{\bar{a}}_{nb}p_{nb}^{\prime }+b$$

where

(7) $${\bar{a}}_{nb}=\frac{\rho_{f}\left(\Omega_{p}+\Omega_{nb}\right)\vec{A}\cdot \vec{A}}{\left(a_{p}+a_{nb}\right)ds\vec{A}\cdot {\vec{e}}_{s}}$$

(8) $${\bar{a}}_{P}=\sum_{nb}{\bar{a}}_{nb}$$

$p^{\prime }$is the pressure correction; *b* is a source term that includes all terms not included in the first and second terms of Eq. (6); $a_{p}$ and $a_{nb}$are point coefficients contained in the discretised Navier-Stokes equation, Eq. (5); $\Omega_{p}$ is volume of cell *P* and $\Omega_{nb}$ is the volumes of cell neighbour *nb*; $\vec{A}$, ${\vec{e}}_{s}$, and dsare the face area vector, the unit vector from cell *P* to cell neighbour *nb*, and the distance from cell *P* to *nb*, respectively.

Based on the SIMPLE algorithm, Eq. (5) was solved using estimated values as initial values for pressure and velocity. The pressure and velocity obtained from the solution of Eq. (5) were updated using the pressure correction obtained from Eq. (6). Eq. (5) was then solved again using the updated value; the procedure was repeated until convergence was reached.

## 3 Interpolation to cell faces

To find the solution of the discretised Navier-Stokes equation (Eq. (4)), face average values for flow variable φ are required to compute the convective flux across the cell faces. Eight methods for calculating the face average values of φ are presented in this section.

### 3.1 Linear interpolation between cell centres

This method is based on the one-dimensional interpolation approach along the line connecting cells located to the left and right of the surface where the face value of φ is to be obtained. Assuming the face centre is midway between adjacent cell centres, the face value of φ is expressed as

(9) $$\varphi_{f}\approx \frac{\varphi_{L}+\varphi_{R}}{2}$$

**Figure 2:**
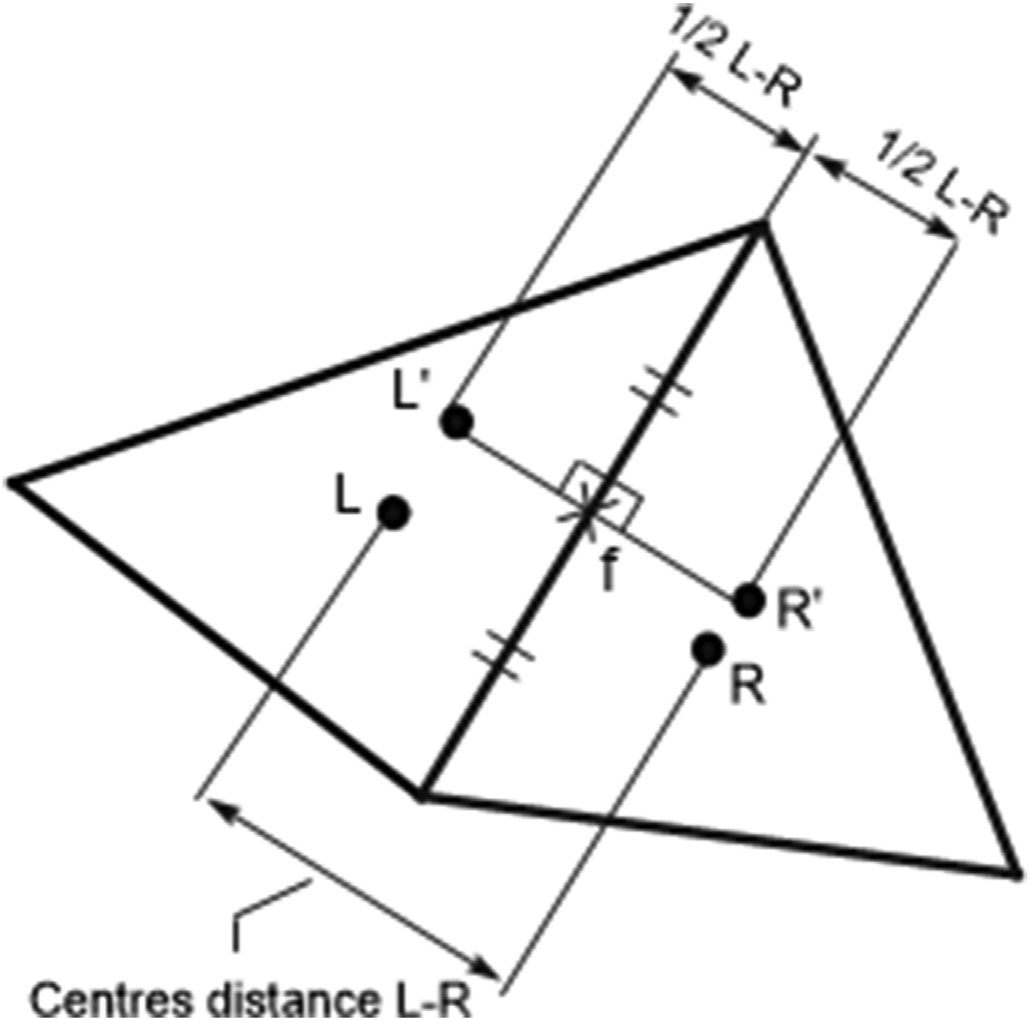
Linear interpolation stencil for interpolation to faces.

This interpolation is only truly second-order accurate for the special case of equilateral triangular cells, where face centres are co-linear and equidistant from the cell centres *L* and *R,* as shown in Figure 2.

### 3.2 Spatially correct linear interpolation between cell centres

The linear interpolation method gives the value of φ at the point of intersection of the surface with a line connecting the cells to the left and right of the surface. If the intersection point is not at the centre of the surface, there is an increased error in the surface integral of the convective flux in Eq. (3). To correct these errors, a spatial correction is necessary.

A spatially correct linear interpolation scheme can be constructed using auxiliary points $L^{\prime }$ and $R^{\prime }$, as shown in Figure 2. These auxiliary points are related to cell centres *L* and *R*, but are located on the normal bisector of the face, equidistant from the face centre, such that the distance between $L^{\prime }$ and $R^{\prime }$ is equal to the distance between *L* and *R*; φ at the auxiliary points $L^{\prime }$ and $R^{\prime }$ is determined by Taylor series expansions about cell centres *L* and *R,* respectively. The sequence of operations is

(10) $$\varphi_{L^{\prime }}\approx \varphi_{L}+{\left(\frac{\partial \varphi }{\partial x}\right)}_{L}\text{Δ}x_{LL^{\prime }}+{\left(\frac{\partial \varphi }{\partial y}\right)}_{L}\text{Δ}y_{LL^{\prime }}$$

(11) $$\varphi_{R^{\prime }}\approx \varphi_{R}+{\left(\frac{\partial \varphi }{\partial x}\right)}_{R}\text{Δ}x_{RR^{\prime }}+{\left(\frac{\partial \varphi }{\partial y}\right)}_{R}\text{Δ}y_{RR^{\prime }}$$

(12) $$\varphi_{f}\approx \frac{\varphi_{L^{\prime }}+\varphi_{R^{\prime }}}{2}$$

It is suggested that the additional error associated with a two-stage process may not be large, as the distances $LL^{\prime }$ and $RR^{\prime }$are generally small compared to the cell dimensions.

An alternative spatially correct central difference scheme can be obtained by averaging the face value given by a Taylor series expansion about cell centre *L*, with a value given by a similar Taylor series expansion about cell centre *R*:

(13) $$\begin{array}{l} \varphi_{f}\approx \frac{1}{2}\left(\varphi_{L}+{\left(\frac{\partial \varphi }{\partial x}\right)}_{L}\text{Δ}x_{Lf}+{\left(\frac{\partial \varphi }{\partial y}\right)}_{L}\text{Δ}y_{Lf}\right)+\\ \;\frac{1}{2}\left(\varphi_{R}+{\left(\frac{\partial \varphi }{\partial y}\right)}_{R}\text{Δ}x_{Rf}+{\left(\frac{\partial \varphi }{\partial y}\right)}_{R}\text{Δ}y_{Rf}\right) \end{array}$$

### 3.3 Approximate QUICK

**Figure 3:**
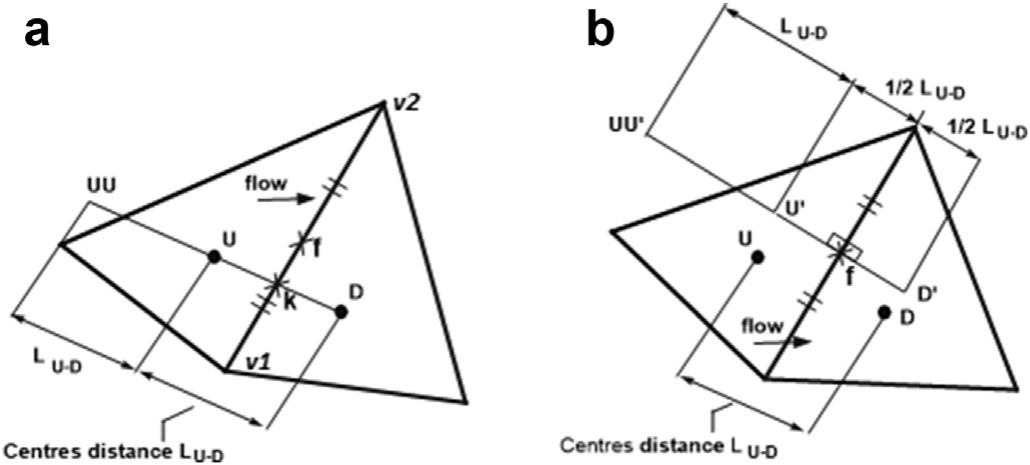
Approximate QUICK stencils for interpolation to faces. (a) Standard scheme; (b) Spatially correct scheme.

The QUICK scheme of Leonard [5] is a popular higher-order upwind-biased face interpolation scheme for structured meshes of quadrilateral cells that uses quadratic interpolation based on two cell centre points on the upstream side of the cell face and one on the downstream side. It is not generally possible to find three such collinear cell centres for unstructured meshes, but Darwish and Moukalled [6] indicate how an approximate equivalent scheme may be constructed by introducing a “fictitious” far upstream point *UU*, such that cell centre *U* is midway between point *UU* and cell centre *D*, as shown in Figure 3a. The value of φ at the fictitious point *UU* is calculated as

(14) $$\varphi_{UU}\approx \varphi_{D}-2{\left(\frac{\partial \varphi }{\partial x}\right)}_{U}\text{Δ}x_{UD}-2{\left(\frac{\partial \varphi }{\partial y}\right)}_{U}\text{Δ}y_{UD}$$

where $\text{Δ}x_{UD}$ and $\text{Δ}y_{UD}$ are the components of the vector displacement from point *U* to point *D*. For an approximate QUICK scheme, the face value of φ is expressed as

(15) $$\varphi_{f}\approx \frac{1}{8}\left(3\varphi_{D}+6\varphi_{U}-\varphi_{UU}\right)$$

The method is denoted here as “approximate QUICK”, as the spacing and collinearity errors described for the approximate linear interpolation scheme are still present.

### 3.4 Spatially correct approximate QUICK

A “spatially correct" version of the Darwish and Moukalled QUICK scheme can be made using auxiliary points $U^{\prime }$and $D^{\prime }$, as shown in Figure 3b. The φvalues at these auxiliary points are determined as described in the previous sub-section; the method follows Eq. (14) and Eq. (15), replacing subscripts *UU*, *U,* and *D* with *U*$U^{\prime }$, $U^{\prime }$, and $D^{\prime }$.

(16) $$\varphi_{f}\approx \frac{1}{8}\left(3\varphi_{D^{\prime }}+6\varphi_{U^{\prime }}-\varphi_{UU^{\prime }}\right)$$

### 3.5 Polynomial interpolation

φ at the face centre can be estimated by assuming that φ is distributed as a three-order polynomial along the line connecting cell centres located to the left and right of the face.

(17) $$\varphi \left(r\right)=a_{0}+a_{1}s+a_{2}s^{2}+a_{3}s^{3}$$

where *s* represents a local coordinate axis along the line from the left cell to the right cell of the face under consideration; *a*_*0*_, *a*_*1*_, *a*_*2*_, and *a*_*3*_ are unknown constants.

The four unknown constants can be obtained using φ and ∂φ∂s from the cells to the left and the right of the face under consideration. Thus, φ at the face centre can be expressed as Eq. (18).

(18) $$\varphi_{f}=\frac{\varphi_{L}+\varphi_{R}}{2}+\frac{r_{LR}}{8}\left(\nabla \varphi_{L}\cdot \vec{s}-\nabla \varphi_{R}\cdot \vec{s}\right)$$

Subscripts *L* and *R* refer to cells to the left and the right of the face; $r_{LR}$ and $\vec{s}$ are the distance from cell *L* to cell *R* and the unit vector from cell *L* to cell *R*, respectively.

### 3.6 Linear interpolation between end point vertices

An alternative two-stage interpolation method is to interpolate first from cell centres to cell vertices and obtains face centre values of φ by linear interpolation between face end or corner vertices. In two dimensions:

(19) $$\varphi_{f}\approx \frac{\varphi_{v1}+\varphi_{v2}}{2}$$

where $\varphi_{v}$ is the vertex value of φ. The accuracy of this non-biased approach is limited primarily by the accuracy of the first stage interpolation of the vertices.

### 3.7 Taylor series expansion about upstream cell centre

**Figure 4:**
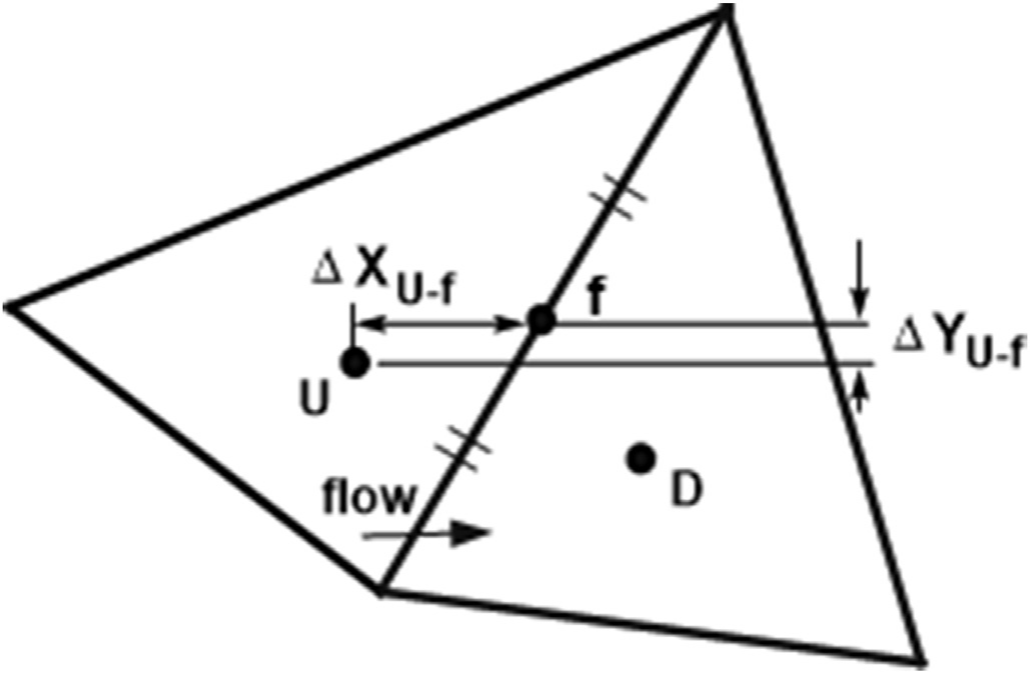
Stencil for Taylor series expansion about upstream cell centre.

The classic second-order upwind scheme of Barth and Jespersen [4] approximates the face centre value of φ using a Taylor series expansion about the cell centre point on the upstream side of the face:

(20) $$\varphi_{f}\approx \varphi_{u}+{\left(\frac{\partial \varphi }{\partial x}\right)}_{U}\text{Δ}x_{Uf}+{\left(\frac{\partial \varphi }{\partial y}\right)}_{U}\text{Δ}y_{Uf}$$

where $\text{Δ}x_{Uf}$and $\text{Δ}y_{Uf}$ are the components of the displacement vector from cell centre *U* to face centre *f*, as shown in Figure 4. The approximation is evidently dependent upon the accuracy of the partial derivatives at cell centre *U*, but second-order accuracy is obtained with accurate first-order derivatives.

### 3.8 Laplacian interpolation to face centres

A Laplacian of a variable can be estimated using a pseudo-Laplacian as proposed by Holmes and Connel [18]. Applying the pseudo-Laplacian to determine the Laplacian of flow variable φ on the face centre of the unstructured mesh, the following equation is obtained:

(21) $$L{\left(\varphi \right)}_{f}=\sum_{i=1}^{N}w_{i}\left(\varphi_{i}-\varphi_{f}\right)$$

where *i* represents cell centres $nb_{1}$, $nb_{3}$*,* and *P* located upstream of the face *f*, and a cell $nb_{2}$ downstream of the face, as shown in Figure 1. The use of upstream cells to calculate $\varphi_{f}$ provides an upstream bias condition that prevents oscillations in the area around locations with a strong gradient of φ [5].

If φis linearly distributed, the Laplacian of φ should be zero. Thus, Eq. (21) can be rearranged to solve for $\varphi_{f}$.

(22) $$\varphi_{f}=\frac{\sum_{i=1}^{N}w_{i}\varphi_{i}}{\sum_{i=1}^{N}w_{i}}$$

The weights $w_{i}$ are chosen to achieve the stability of the numerical, finite volume, solution such that Eq. (22) meets the positivity requirements [5]. The positivity requirements are satisfied if $w_{i}$ is positive. Following Holme and Connel [18], $w_{i}$ is defined in Eq. (23).

(23) $$w_{i}=1+\text{Δ}w_{i}$$

where $\text{Δ}w_{i}$ is of the form

(24) $$\text{Δ}w_{i}=\beta_{x}\left(x_{i}-x_{f}\right)+\beta_{2}\left(y_{i}-y_{f}\right)$$

$x_{i}$, $y_{i}$ are rectangular coordinates of cells $nb_{1}$, $nb_{2}$, $nb_{3}$, and P; $x_{f}$, $y_{f}$ are rectangular coordinates of face centre *f*; $\beta_{x}$ and $\beta_{y}$ are expressed as

(25) βx=IxxRy−IyyRxIxxI−yyIxy2

(26) βy=IxyRx−IxxRyIxxI−yyIxy2

where

(27) $$R_{x}=\sum_{i=1}^{N}\left(x_{i}-x_{f}\right)$$

(28) $$R_{y}=\sum_{i=1}^{N}\left(y_{i}-y_{f}\right)$$

(29) $$I_{xx}={\sum_{i=1}^{N}\left(x_{i}-x_{f}\right)}^{2}$$

(30) $$I_{yy}={\sum_{i=1}^{N}\left(y_{i}-y_{f}\right)}^{2}$$

(31) $$I_{xy}=\sum_{i=1}^{N}\left(x_{i}-x_{f}\right)\left(y_{i}-y_{f}\right)$$

## 4 Comparison of methods for interpolation to faces

In this section, the accuracy of individual component algorithms for interpolation are tested. Kovasznay flow [15] and potential solutions of flow past a circular cylinder, modelled as a combination of source and sink of equal strength, were used as the analytical solution benchmarks [17]. These were chosen over purely arbitrary analytical functions to be more representative of typical high-Reynolds number flows.

Adopting the test procedure used by Syrakos et al. [19], accuracy of the individual interpolation algorithm was considered in isolation, rather than the effect of the interpolation algorithm when used in a complete Navier-Stokes solver. For this purpose, a typical triangular unstructured mesh was overlaid on the flow domain. The procedure in each case was to evaluate cell centre values of a variable φ, typically a velocity component, from analytical solution, then to use these values only as data for the algorithm under test, to calculate interpolated values of φ at cell faces. Exact values at these locations are available from the analytic solution and so the error in the value computed via the test algorithm may be determined at each point. The *L1* norm of these errors, taken over the whole domain except for boundary values, may be used as a comparative measure of the algorithm accuracy.

By varying the mean mesh spacing, the order of accuracy of the algorithm can be estimated. The standard meshes were produced according to Delaunay triangulation procedure using Ansys R2 software, with equally cell face area specified on the boundaries. The standard meshes was made so that they have maximum equiangle skewness of 0.35, with an average of 0.1. To simulate the effects of mesh distortion, ‘randomised’ meshes were also produced, with mesh vertex coordinates randomly perturbed by a fraction of the mean cell size. The distorted meshes have a maximum and average equiangle skewness of 0.95 and 0.7, respectively.

**Figure 5:**
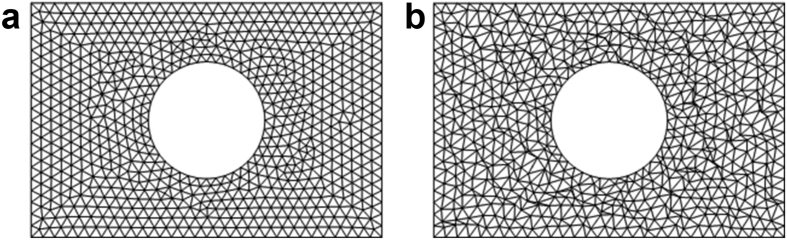
Mesh used for potential flow past a circular cylinder. (a) Standard test mesh; (b) Distorted test mesh.

**Figure 6:**
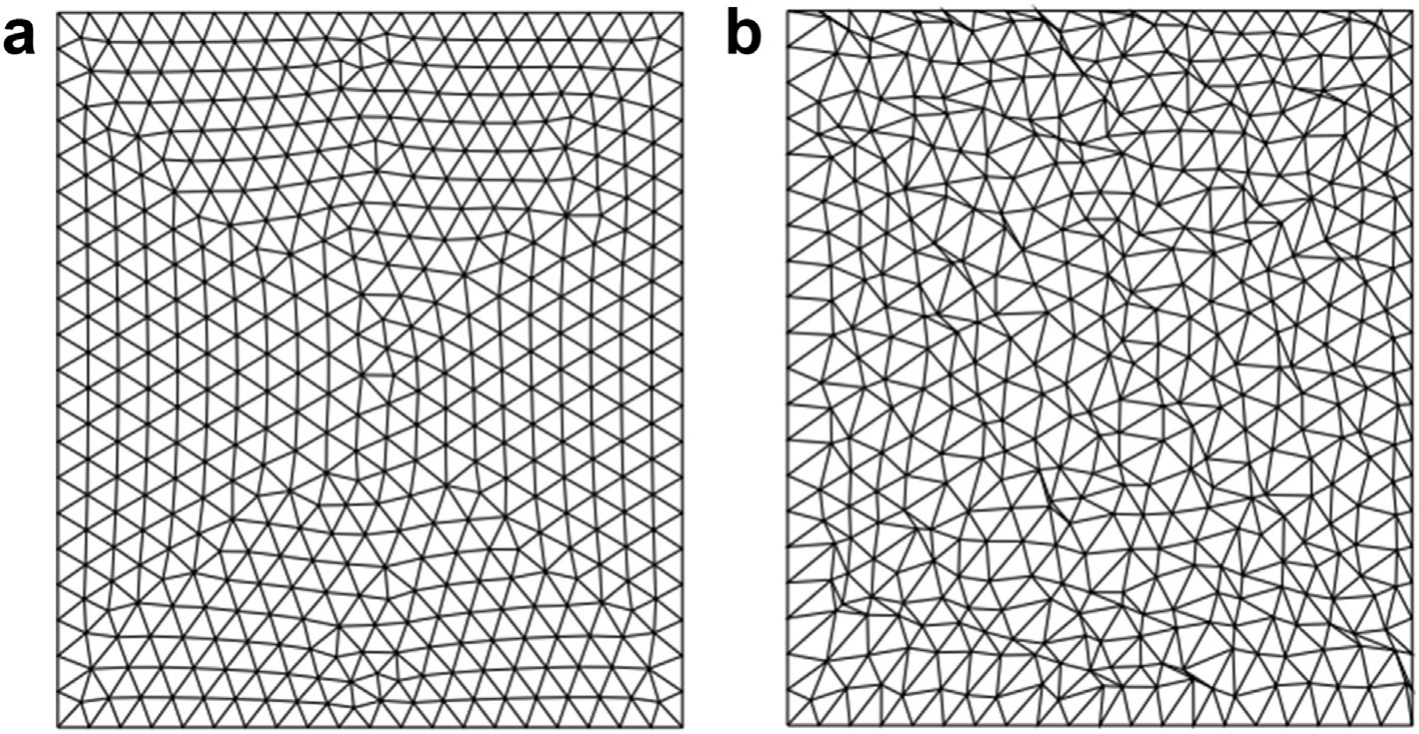
Mesh used for Kovasznay flow. (a) Standard test mesh; (b) Distorted test mesh.

The standard and distorted meshes of triangular cells with an average area of 0.015 m^2^, 0.0017 m^2^, and 0.00015 m^2^ are used. The standard and distorted meshes used for the potential flow past a circular cylinder test case are shown in Figure 5; the mesh used in the Kovasnay flow test case is shown in Figure 6. Both meshes are the same as meshes used by Tasri [20].

**Figure 7:**
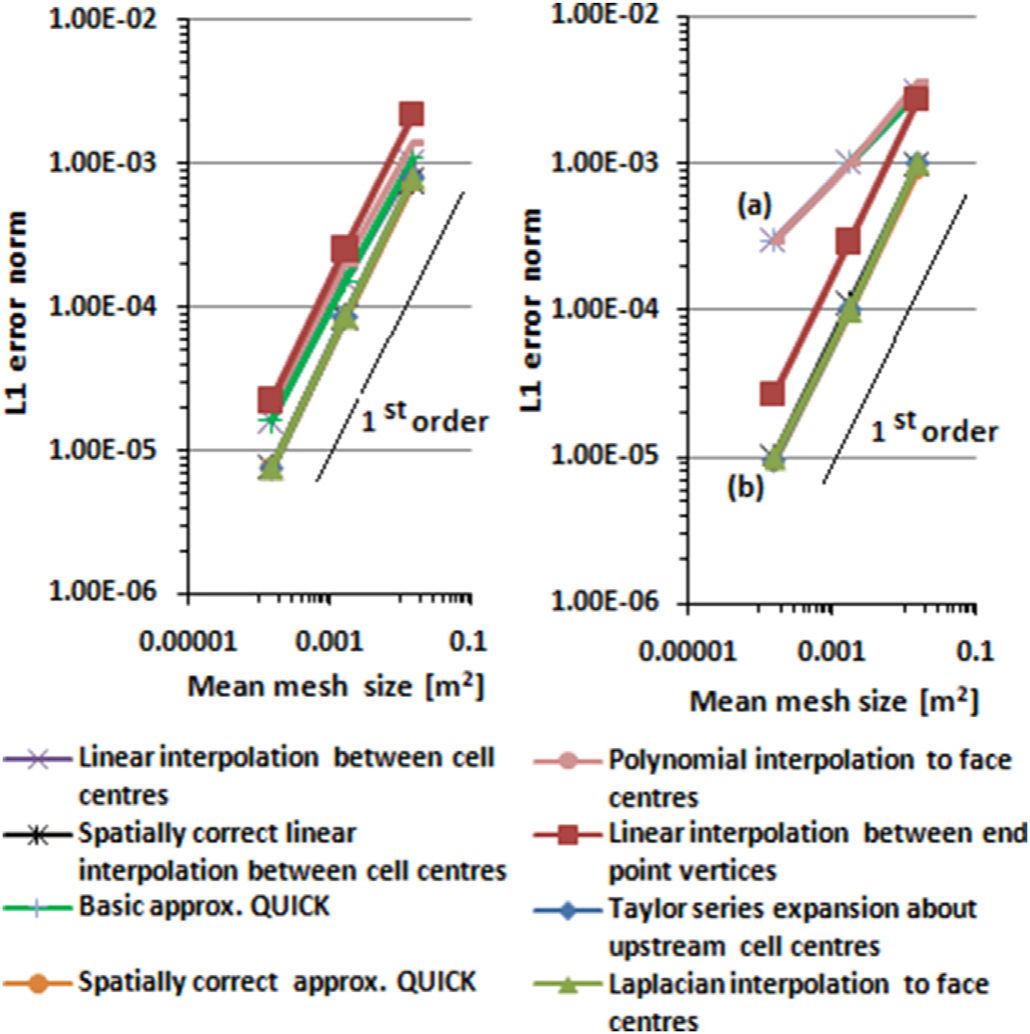
Interpolation errors of cell faces for potential flow past a circular cylinder.

**Figure 8:**
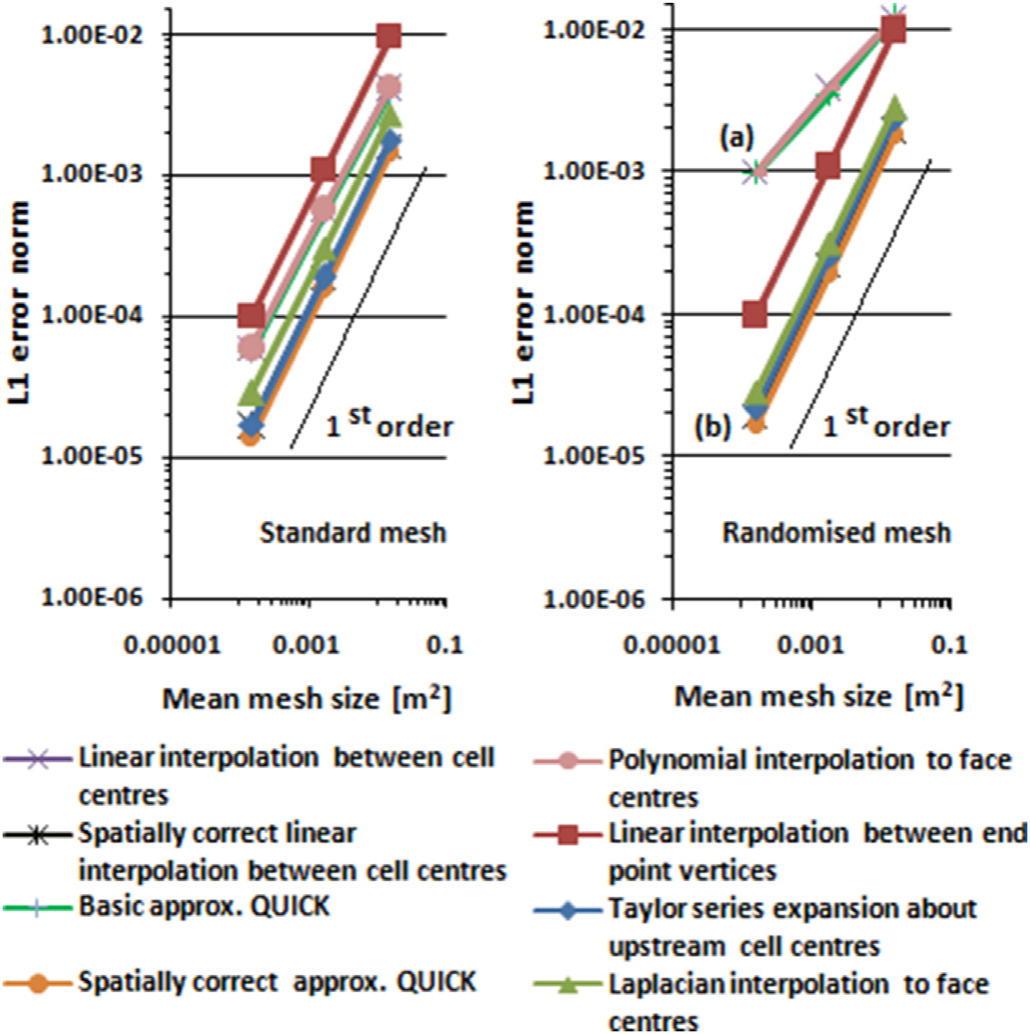
Interpolation errors of cell faces for Kovasznay flow.

*L1* error norms for potential flow past a circular cylinder and Kovasznay flow are shown in Figure 7 and Figure 8, respectively. Line (a) in Figure 7 and Figure 8 is a superimposition of linear interpolation between cell centres, basic approximate QUICK, and polynomial interpolation to face centre. Line (b) is a superimposition of Laplacian interpolation, Taylor series expansion interpolation about upstream cell centres, spatially correct approximate QUICK, and spatially correct linear interpolation between cell centres.

**Figure 9:**
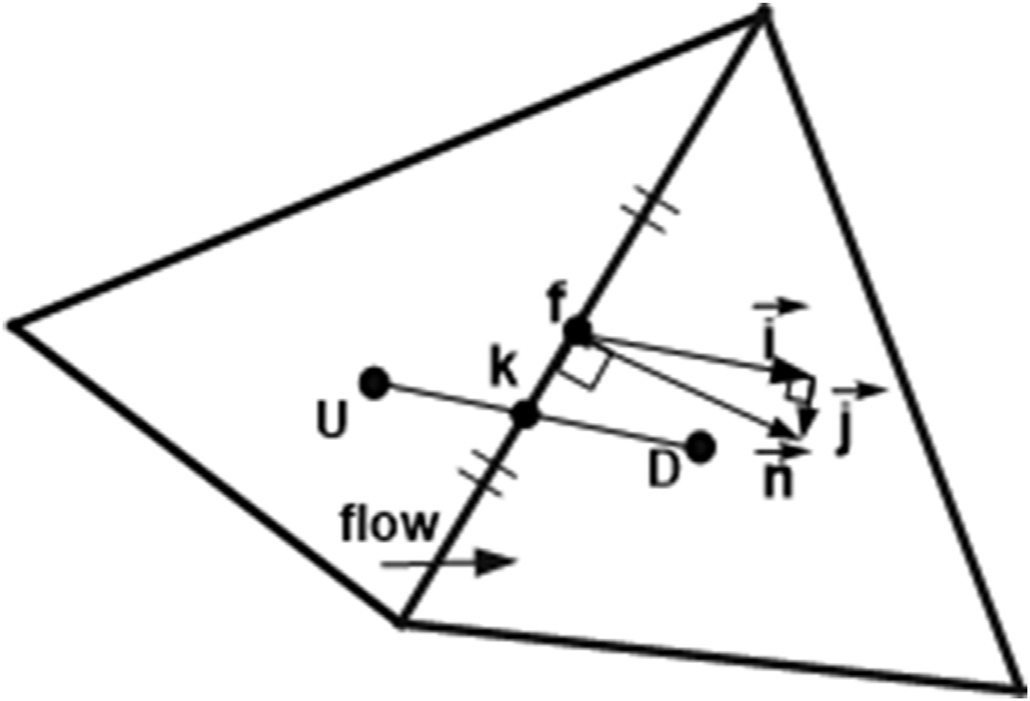
Non-conjunctionality and non-orthogonality of face.

Figure 7 and Figure 8 show that the interpolations based on one-dimensional interpolation along lines connecting cells to the right and the left of the face under consideration (linear interpolation between cell centres, basic approximate QUICK, and polynomial interpolation to face centres) only have first-order accuracy in standard unstructured mesh, and less than first-order accuracy in distorted mesh, although the interpolations formally have second- and third-order accuracy. *L1* errors of the interpolations in distorted mesh were greater than in standard mesh. The lower order of accuracy and higher *L1* errors in distorted mesh were most likely caused by non-conjunctionality and non-orthogonality conditions of finite volume mesh. Non-conjunctionality is a condition where the intersection point between the control volume face and the line connecting cells to the right and left of the face are not located at the face centre, as shown in Figure 9. Non-orthogonality is a condition where the line connecting cells to the right and left of the control volume face are not normal to the face. In the case of non-conjunctionality, the interpolation point is not located at the face centre but at the intersection of the face and the line connecting cells to the right and left of the face. If data at the interpolation point is used to estimate the surface integral of the convective term on the left side of Eq. (4), the accuracy of the surface integral will be less than second-order, as second-order midpoint surface integration requires data at the face centre.

To determine how the error affects the accuracy of the finite volume solution, φ at the face centre *f* is written as a reconstruction of φ at the interpolation point *k* (Figure 9) using Taylor series expansion:

(32) $$\varphi_{f}=\varphi_{k}+\nabla \varphi_{k}\cdot \vec{r}$$

where $\vec{r}$is the Euclidean vector from interpolation point k to the face centre f. $\varphi_{f}$ and $\varphi_{k}$ are φ at the face centre and the interpolation point, respectively. If φ at the face centre in Eq. (4) is estimated with φ at the interpolation point k in Eq. (32), the result is:

(33) $$\sum_{m}{\left(\rho_{f}\varphi_{f}{\vec{V}}_{f}\right)}_{m}\cdot \vec{n}A_{m}-\sum_{m}{\left(\rho_{f}\nabla \varphi_{k}\cdot \vec{r}\right)}_{m}{\left({\vec{V}}_{f}\cdot \vec{n}\right)}_{m}A_{m}=\sum_{m}{\left(\mu_{f}\nabla \varphi_{f}\right)}_{m}\cdot \vec{n}A_{m}+q\Omega$$

The second term on the left side of Eq. (32) is an estimation error. The error has a form similar to the diffusive term of the governing equation. Error terms that similar to diffusive terms tend to reduce the gradient of φ from the value it should be.

Interpolation errors in the distorted mesh can also be caused by non-orthogonality of the face and the line connecting cells to the left and right of the face. Multiplication of the gradient flow variable on the control volume surface with the vector normal to the surface, as found in the diffusive term of the discretised governing equation (Eq. (4)), can be expressed as

(34) $$\nabla \varphi \cdot \vec{n}\approx \nabla \varphi \cdot \vec{i}+\nabla \varphi \cdot \vec{j}$$

Vector $\vec{i}$ in Eq. (34) is the component of the normal vector $\vec{n}$ in the direction parallel to the line connecting cells on the left and right sides of the surface, as shown in Figure 9; $\vec{j}$ is the component of vector $\vec{n}$ in the direction perpendicular to $\vec{i}$. If the line connecting the cells to the left and right of the surface is orthogonal to the surface, the second term is zero. The second term is often ignored because it can produce unboundedness of discretised equation. However, ignoring this term reduces the accuracy of the calculation.

Applying spatial correction to basic approximate QUICK and linear interpolation between cell centres to correct the non-conjunctionality and non-orthogonality, *L1* error is reduced and order of accuracy is increased from less than first order to first-order. The spatially corrected versions of basic approximate QUICK and linear interpolation between cell centres have lower *L1* errors and higher orders of accuracy than the non-spatially corrected versions, as shown in Figure 7 and Figure 8.

Unlike one-dimensional interpolation along the line connecting cells to the right and left of the face, multi-dimensional interpolation, the Laplacian interpolation, and Taylor series expansion about an upstream cell centre can provide interpolation at the surface centre to minimize the conjunctionalty error. Figure 7 and Figure 8 show that the tested multi-dimensional interpolations are better than the one-dimensional based interpolation, especially with a distorted mesh. The multi-dimensional interpolations have first-order accuracy on standard unstructured mesh and remain first-order accurate when used in distorted mesh. The *L1* error on the standard mesh is the same as the *L1* error on the distorted mesh; the multi-dimensional interpolations are unaffected by the quality of the mesh.

Interpolation from vertices is not affected by the non-conjunctionality conditions as the error of this interpolation is similar for standard and distorted mesh. The error of this interpolation is mainly from first-step interpolation from the cell centre to vertices.

The interpolation from vertices has the highest *L1* error in standard unstructured mesh, but in distorted mesh, this interpolation performs better than standard one-dimensional interpolation along the line connecting neighbour cells. Linear interpolation between cell centres, basic approximate QUICK, and polynomial interpolation to face centre have the greatest *L1* error in distorted unstructured mesh. Laplacian interpolation to the face centre, spatially correct interpolation between cell centres, spatially correct approximate QUICK, and Taylor series expansion about upstream cell centres have the lowest *L1* error in standard and distorted unstructured meshes.

Considering the simplicity of the computer code needed for interpolation methods, the accuracy of the interpolations, and the positivity requirements of the discretized governing equation [5], the Taylor series expansion about an upstream cell centre is the best choice for interpolation from cell centre to face centre in standard or distorted unstructured mesh.

All interpolations were derived and tested for 2-D flows. Nevertheless, 3-D versions of the interpolations can be derived in a similar manner. The cause of the interpolation errors is the same in 3-D and 2-D. Thus, conclusions obtained in 2-D cases are also applicable to 3-D cases.

## 5 Discussion and conclusions

After testing a range of compact-stencil interpolation algorithms in this study, the following conclusions can be drawn:

1. Formally second- and third-order accurate interpolations based on one-dimensional interpolation along the line connecting cell centres to the left and the right of the face under consideration only have first-order accuracy on standard unstructured mesh. These interpolations have less than first-order accuracy on distorted unstructured mesh. *L1* errors in distorted unstructured mesh are greater than in standard unstructured mesh.
2. *L1* errors and the degree of accuracy of formally second- and third-order accurate interpolations based on one-dimensional interpolation along the line connecting cell centres to the left and the right of the face under consideration can be improved with spatial correction.
3. The formally second-order multi-dimensional interpolations not based on one-dimensional interpolation along the line connecting neighbour cells of the face under consideration have first-order accuracy on standard and distorted unstructured mesh; there were no differences in *L1* errors for standard and distorted unstructured mesh.
4. Among the tested methods, linear interpolation between end vertices has the greatest *L1* error in standard unstructured mesh. Polynomial interpolation, standard QUICK, and linear interpolation between cell centres have the highest *L1* error in distorted unstructured mesh. Spatially correct QUICK, spatially correct linear interpolation between cell centres, Laplacian interpolation to face centres, and Taylor series expansion about an upstream cell have the smallest *L1* error in standard and distorted unstructured mesh.
5. Based on simplicity and accuracy, Taylor series expansion about an upstream cell centre is the best choice for interpolation from cell centre to face centre for unstructured mesh.

## Declarations

### Author contribution statement

A. Tasri: Conceived and designed the experiments; Performed the experiments; Analyzed and interpreted the data; Contributed reagents, materials, analysis tools or data; Wrote the paper.

A. Susilawati: Contributed reagents, materials, analysis tools or data; Wrote the paper.

### Funding statement

This research did not receive any specific grant from funding agencies in the public, commercial, or not-for-profit sectors.

### Data availability statement

Data included in article/supplementary material/referenced in article.

### Declaration of interests statement

The authors declare no conflict of interest.

### Additional information

No additional information is available for this paper.
